# *EGFR* Mutation-Harboring Lung Cancer Cells Produce CLEC11A with Endothelial Trophic and Tumor-Promoting Activities

**DOI:** 10.3390/cancers14051356

**Published:** 2022-03-07

**Authors:** Tzu-Yin Lin, Chi-Hwa Yang, Hsiao-Chin Chou, Chun-Mei Cheng, Ya-Wen Liu, Jiz-Yuh Wang, Li-Rung Huang, Shih-Feng Tsai, Shiu-Feng Huang, Yi-Rong Chen

**Affiliations:** 1 Institute of Molecular and Genomic Medicine, National Health Research Institutes, Zhunan 35053, Taiwan; 040125@nhri.edu.tw (T.-Y.L.); 920507@nhri.org.tw (C.-H.Y.); 970923@nhri.org.tw (H.-C.C.); 050202@nhri.org.tw (C.-M.C.); yawen342@gmail.com (Y.-W.L.); jizyuhwang@cc.kmu.edu.tw (J.-Y.W.); lrhuang@nhri.edu.tw (L.-R.H.); petsai@nhri.edu.tw (S.-F.T.); sfhuang@nhri.edu.tw (S.-F.H.); 2 Graduate Institute of Medicine, College of Medicine, Kaohsiung Medical University, Kaohsiung 80708, Taiwan; 3 Institute of Genetics, Genome Research Center, National Yang-Ming Chiao-Tung University, Hsinchu 30010, Taiwan; 4 Department of Anatomic Pathology, Linko Chang Gung Memorial Hospital, Chang-Gung University, Taoyuan 33302, Taiwan

**Keywords:** CLEC11A, lung cancer, angiogenesis, endothelial cell, microenvironment

## Abstract

**Simple Summary:**

Tumor angiogenesis is an important step in the progression of solid tumors. Understanding the mechanisms involved in tumor vasculature formation is critical for improving anti-angiogenic strategies. In this study, we reported that *EGFR* mutation-containing lung cancer cells produced CLEC11A with endothelial trophic and tumor-promoting activities. CLEC11A could be a novel factor involved in tumor angiogenesis.

**Abstract:**

The formation of new blood vessels in solid tumors is regulated by various endothelial trophic factors. We identified that CLEC11A, an extracellular C-type lectin, was over-expressed in lung cancer cell lines harboring mutated *EGFR*. CLEC11A expression was also frequently elevated in lung adenocarcinoma (LAC) tissues with *EGFR* mutation. CLEC11A-expressing H1299 cells formed larger tumors in nude mice than did the control cells. The CLEC11A-expressing tumors contained more CD31-positive cells, suggesting that they had a higher angiogenic activity. CLEC11A per se did not induce blood vessel formation, but enhanced angiogenesis triggered by VEGF-A or basic FGF in vivo. Additionally, the expression of small hairpin RNA against CLEC11A (shCLEC11A) in HCC827 LAC cells suppressed their tumorigenic ability. Purified CLEC11A exhibited a chemotactic ability, which is dependent on its integrin-binding RGD and LDT motifs, toward endothelial cells. This chemotactic activity was not affected by the presence of a VEGFR inhibitor. Conditioned medium produced by HCC827-shCLEC11A cells had diminished chemotactic ability toward endothelial cells. CLEC11A treatments increased the levels of active integrin β1 that were not associated with activation of focal adhesion kinases in endothelial cells. Our results indicated that CLEC11A was a factor of angiogenic potential and was involved in lung cancer tumorigenesis.

## 1. Introduction

Angiogenesis, the formation of new blood vessels from pre-existing endothelial cells, is regulated by the balance of pro-angiogenic and anti-angiogenic factors present in a tissue [1]. In solid tumors, including lung cancer, the mechanisms involved in angiogenesis have been considered as therapeutic targets, because angiogenesis is essential for primary tumor growth and for metastasis in advanced cancers [1,2]. It is known that hypoxia in the tumor mass, which is a major cause of angiogenesis, leads to the overproduction of pro-angiogenic factors [1,3]. The best characterized family of angiogenic factors is the vascular endothelial growth factor (VEGF) family, which consists of VEGF-A through D and placenta growth factor (PlGF, also named phosphatidylinositol glycan anchor biosynthesis class F (PIGF)) [1,2]. VEGFs binds to a family of transmembrane receptor tyrosine kinases (RTKs) called VEGF receptors (VEGFR1-3). VEGF-A, mainly responsible for vessel formation in adult tissues, binds to VEGFR-1 with higher affinity; however, it has been reported that VEGFR-2, which is the primary receptor involved in endothelial cell proliferation and migration, mediates VEGF-A-induced angiogenesis in cancers [2]. In addition to VEGF family members, other factors (such as fibroblast growth factors (FGFs)) and chemokines have also been implicated in the processes of normal and tumoral angiogenesis [4]. Tumor angiogenesis has unique characteristics of abnormal and poorly organized vessels with increased permeability. These features may result from the overproduction of various angiogenic factors from the tumor and stromal cells [1,2]. Targeting tumor angiogenesis has been approached through two methods: monoclonal antibodies that block VEGF–VEGFR binding, and small molecule TKIs that inhibit the downstream VEGFR mediated signaling [1].

C-type lectin 11A (CLEC11A, also named stem cell growth factor (SCGF)) is originally identified as an autocrine factor secreted by leukemia cell lines [5]. Full length human CLEC11A protein consists of 323 amino acids with the lectin domain at its carboxyl terminus [6,7]. CLEC11A is also a factor promoting endothelial cell differentiation from AC133-positive progenitor cells [8]. Genetic ablation in mice shows that CLEC11A is also an autocrine factor for osteoblasts, and its abolition causes reduced limb and vertebral bone formation [9]. In contrast to the earlier findings, CLEC11A-null mice do not have obvious defects in hematopoiesis [9]. CLEC11A contains potential integrin-binding motifs (RGD and TDF motifs), and it has been shown that Integrin α11 is the receptor for CLEC11A in osteoblasts [10]. Nevertheless, the RGD motif is not present in the mouse CLEC11A protein [6,10].

Our current study showed that CLEC11A expression was increased in LAC cells expressing mutated EGFR. The elevation of CLEC11A was confirmed in clinical LAC tissues, and high CLEC11A expression in LAC was associated with a worse overall survival. CLEC11A was a chemo-attractant for mouse endothelial cells and enhanced angiogenesis induced by VEGF-A or FGF1. Our results from cellular and animal studies supported that CLEC11A might promote LAC progression through enhancing tumor angiogenesis.

## 2. Materials and Methods

### 2.1. Cell Cultures

NL20-immortalized human bronchial epithelial cells and SVEC4-10 mouse endothelial cells were obtained from the National Health Research Institutes (NHRI) Cell Bank and cultured as described [11,12]. Human lung cancer cell lines (H1299 and its derivatives, CL1-0, H661, H358, H1975, HCC1650, PE089, and HCC827) were cultured as described previously [13,14,15]. NL20, CL1-0, H661, H358, and H1299 cells express wild type (WT) EGFR; H1975 cell line has L858R/T790M double mutations; H1650, PE089, and HCC827 cells have *EGFR* exon 19 deletion (Del). H1299 derivatives expressing CLEC11A were established by transfecting the CLEC11A expression vector into H1299 cells using the lipofectamine 2000 reagent (Thermo Fisher Scientific, Waltham, MA, USA) according to the manufacturer’s instructions, and were subjected to Zeocin (Invitrogen, Waltham, MA, USA) selection for one week to establish permanently transfected cells. HCC827–vector control and HCC827-shCLEC11A cells were established by transfecting an empty pSuper-puromycin vector or a vector encoding a small hairpin RNA against CLEC11A (shCLEC11A) into H1299 cells using the lipofectamine 2000 reagent. The transfected cells were then subjected to puromycin selection (1 μg/mL) for one week to establish permanently transfected cells. The isolation and culture of mouse primary aortic artery endothelial cell (AAE cell) was performed as described previously [16].

### 2.2. Antibodies and Reagents

The anti-CLEC11A monoclonal antibody (Cat# MAB1904) was purchased from R&D System (Minneapolis, MN, USA). The anti-FAK (Cat# sc-1688), anti-integrins α2 (Cat# sc-6586), α5 (Cat# sc-10729), and β1 (Cat# sc-6622), p130-Cas (Cat# sc-20029), and peroxidase-conjugated secondary antibodies were purchased from Santa Cruz (Santa Cruz, CA, USA). The anti-active integrin β1 antibody (Cat# 550531) was obtained from BD Pharmingen (San Diego, CA, USA). The anti-EGFR (Cat#2232), anti-pEGFR (Y1068, Cat# 2234), anti-pFAK (Y576/577, Cat# 3281), and anti-pp130-Cas (Y165, Cat# 4015) were purchased from Cell Signaling Technology (Beverly, MA, USA). The anti-pFAK (Y397; Cat# ab81298) antibody was purchased from Abcam (Cambridge, UK). The anti-actin (Cat# A5441) was purchased from Sigma-Aldrich (St. Louis, MO, USA). Anti-TGFBI (Cat# GTX100744) and anti-tubulin (Cat# GTX62882) antibodies were obtained from GeneTex (Hsinchu, Taiwan). G418 and Zeocin were purchased from Sigma-Aldrich (St. Louis, MO, USA) and Invitrogen (Carlsbad, CA, USA), respectively. VEGF-A 165 (Cat# 292-VE) and basic FGF (Cat# 234-FSE) were purchased from R&D System (Minneapolis, MN, USA).

### 2.3. CLEC11A Expression and Knockdown Vectors

The coding sequence of human CLEC11A was PCR-amplified using a cDNA pool from H1299-EGFR-Del cells as the template. The forward primer sequence is 5′-GCGGGATCCACCATGCAGGCAGCCTGGCTTTTGGGG and the reverse primer is 5′-GCGGTCGACATGAAGGGGAACTCGCAGACGTAG. The amplified DNA fragment was digested by restriction enzymes (Bam HI and Sal I) and was inserted between the Bam HI and Xho I sites of the pcDNA4-His-Myc B vector (Invitrogen). The D63E, D133E, and D63E/D133E mutations of CLEC11A were generated using the site-directed mutagenesis method. The plasmid-expressing small hairpin RNA against human CLEC11A (shCLEC11A) was constructed using the pSuper-retro-puro vector (OligoEngine, Seattle, WA, USA) as a backbone. A 19-nucleotide sequence (5′-TCCTGCCGGAACTGTTGAG) corresponding to the nucleotides 195–213 of human CLEC11A coding region was used for designing the inverted-repeat insertion in the vector according to the manufacturer’s instructions.

### 2.4. Cell Extracts Preparation

Whole cell extract was prepared by suspending 2 × 10^6^ cells in 200 μL of lysis buffer (50 mM Tris (pH 8.0), 150 mM NaCl, 1% Triton X-100, 0.5% deoxycholate, 0.1% SDS, 2 μg/mL leupeptin, 5 μg/mL aprotinin, 1 mM PMSF, 1 mM DTT, and 1 mM Na_3_VO_4_). For His-tagged CLEC11A pull-down assays, the cell extracts were prepared using lysis buffer without deoxycholate and SDS. The cell extracts were kept on ice and vigorously mixed four times with 5 min intervals. The extracts were cleared by centrifugation at 14,000× *g* for 10 min, and the supernatants were collected for further analyses or stored at −80 °C.

### 2.5. Immuno-Blots, Immuno-Fluorescence (IF) Staining, and His-Tagged Protein Pull-Down Assays

Immuno-blotting and IF staining assays were performed as described previously [17]. For pull-down assays, His-tagged CLEC11A and mutant proteins were immobilized on TALON metal affinity resin beads (BD Biosciences, San Jose, CA, USA) and were incubated with 2 mg of SVEC4-10 cell extracts for 2 h at 4 °C with continuous rotation. The complexes were then washed with the lysis buffer (without deoxycholate and SDS) twice, and affinity-purified proteins were boiled in SDS sample buffer and examined by immuno-blotting.

### 2.6. Cell Migration and Statistical Analyses

Transwell migration assays were performed as described previously with modifications [17]. Forty-thousand SVEC4-10 cells were seeded in the top wells (DMEM with 1% FCS) and allowed for Transwell migration for 8 h toward the bottom chambers (DMEM with 1% FCS) with specified attractants. The Transwell membranes were subjected to crystal violet staining, and the data presented were the results of the quantitative data from 15 observed fields of represented experiments. Statistical analyses were performed by two-tailed Student’s *t*-tests.

### 2.7. Matrigel Angiogenesis Assay

C57BL/6 mice were injected subcutaneously in both flanks with 300 μL of growth factor-reduced Matrigel (Corning, Corning, NY, USA) supplemented with basic FGF or VEGF-A (100 ng/mL) in the presence or absence of CLEC11A (1 μg/mL). The control (without CLEC11A) and experimental group (with CLEC11A) were injected into the same mouse in the right and left flank, respectively. Two weeks later, the Matrigel plugs were carefully harvested, weighed, and minced in ice-cold deionized water. The mixtures were kept at 4 °C overnight for hemoglobin extraction. Then, the mixtures were centrifugated at 13,000 rpm for 20 min at 4 °C and the supernatants were collected for hemoglobin measurement by a colorimetric assay using Drabkin’s reagent (Sigma-Aldrich). A total of 250 µL of the reaction mixture was placed in a well of a 96-well plate and the absorbance was measured at 540 nm.

### 2.8. RNA Extraction and Real-Time PCR Quantification of mRNA

H1299 derivative (Vec, EGFR, L858R, Del) cells were maintained in regular culture medium or in serum-free medium for 24 h. Total cellular RNA were extracted using the Trizol reagent (Invitrogen). The quantification of mRNA was conducted by real-time PCR on the LightCycler instrument (Roche Molecular Biochemicals, Indianapolis, IN, USA). The PCR specificity was further verified by checking the amplification products using agarose gel electrophoresis. Real-time PCR was performed using a kit of LightCycler FastStart DNA Masterplus SYBR Green I (Roche, Indianapolis, IN, USA) in a total volume of 20 μL in the LightCycler glass capillaries. The reaction program was: initial heating to 95 °C for 10 min, followed by 45 PCR cycles of heating to 95 °C for 10 s, incubation for 10 s at the annealing temperature specific for the use of primer, and incubation for 25 s at 72 °C. We conducted a melting curve analysis after every real-time PCR to identify PCR product and to detect the possible presence of contaminating products. We also quantified the transcripts of the *β-Actin* gene, and each sample was normalized on the basis of its β-actin mRNA level. The relative quantification was determined using the comparative CT method.

### 2.9. Experimental Animals

Eight-week-old female BALB/c nude mice or C57BL/6 mice were purchased from the National Laboratory Animal Center, Taiwan. The animals had access to food and water *ad libitum*. Experimental procedures using animals were approved by the Institutional Animal Care and Use Committees of the National Health Research Institutes. The nude mice were injected subcutaneously with H1299-Vec, H1299-CLEC11A, HCC827-Vec, or HCC827-shCLEC11A cells. Each injection contained 2 × 10^6^ cells suspended in 150 μL of RPMI 1640 medium with 30% of Matrigel. At four to five weeks post-injection, the tumors were harvested, weighed, and processed for immunohistochemistry or Western blot analyses. C57BL/6 mice were injected subcutaneously with Lewis lung carcinoma (LL/2) cells. Each injection contained 1 × 10^6^ cells suspended in 150 μL of RPMI 1640 medium with 30% of Matrigel with or without CLEC11A (1 μg/mL). At two weeks post-injection, the tumors were harvested, weighed, and processed for further analyses.

### 2.10. LAC Tissue Sections and Immunohistochemical (IHC) Staining

Parafilm-embedded LAC tissues sections were obtained from Chang Gung Memorial Hospital (CGMH) tissue bank. The study protocol was reviewed and approved by the Institutional Review Boards of CGMH and NHRI. The *EGFR* mutation status of the LAC tissues was determined as previously described [18]. IHC staining was performed at the Pathology Core Laboratory, National Health Research Institutes. The assays were performed on 4 μm thick formalin-fixed paraffin-embedded tissue sections. The sections were deparaffinized twice in xylene for 10 min and twice in ethanol for 2 min, and then placed in 10 mM citric buffer solution (pH 6.0) and heated at 95 °C for 15 min. The samples were washed once with PBS and the endogenous peroxidase activity was blocked by 3% H_2_O_2_ for 5 min. The slides were rinsed twice with PBS and then incubated with an anti-CLEC11A monoclonal antibody (MAB1904; R&D System) at 1:50 dilution for 2 h at room temperature. The slides were washed three times with PBS, and then incubated with an anti-mouse antibody conjugated with peroxidase (1:500; Santa Cruz) for 1 h. The slides were then washed with PBS three times and incubated with 3,3′ Diaminobenzidine (DAB) chromogen for 10 min. After the reaction, the slides were washed with water three times and counterstained with hematoxylin.

### 2.11. Clinical Data Analyses

The data of the LAC, breast cancer (BRCA), as well as head and neck cancer (HNSC) and their respective normal tissues in The Cancer Genome Atlas (TCGA) database were analyzed for *CLEC11A* mRNA levels through the Boxplot program in Gene Expression Profiling Interactive Analysis (GEPIA). To analyze the *CLEC11A* expression in the LAC tissues with or without a mutation in *EGFR* and *K-Ras* genes, the *CLEC11A* expression levels of LAC patients were retrieved through the OncoLnc server and were sub-grouped and analyzed according to their *EGFR* and *K-Ras* mutation records retrieved from the Genomic Data Commons (GDC) Data Portal, NCI. The correlation of the *CLEC11A* expression levels (RNA sequencing data) and the survival of LAC patients was examined through the Kaplan–Meier plotter.

## 3. Results

### 3.1. CLEC11A Levels Are Higher in Lung Cancer Cells Expressing Mutated EGFR

**Figure 1:**
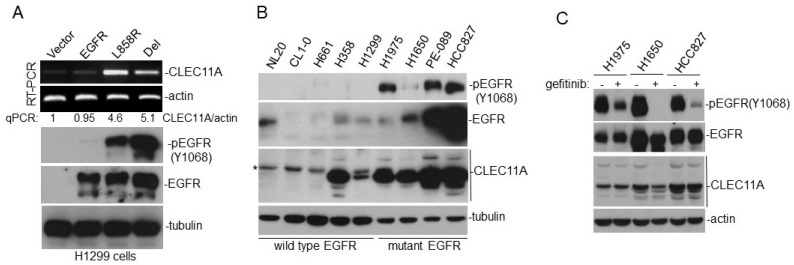
CLEC11A expression is increased in lung cancer cell lines expressing mutated EGFR. (**A**) H1299 cell derivatives were examined for CLEC11A mRNA expression using conventional RT-PCR and quantitative PCR assays. Levels of EGFR and phosphor-EGFR were detected by immune-blotting using specific antibodies. (**B**) NSCLC cell lines with endogenous wild type or mutated EGFR were examined for the indicated proteins by immune-blotting. The asterisk indicates a cross-reactive protein recognized by the antibody. (**C**) H1975, H1650, and HCC827 cells were treated with or without 1 mM of gefitinib for 2 days and the levels of indicated proteins were examined by immune-blotting.

EGFR proteins with a mutation within the kinase domain are constitutively activated in LAC cells [15]. In searching for genes differentially expressed in lung cancer cells with mutated EGFR, we performed microarray analyses comparing the mRNA expression profiles between H1299 NSCLC cells permanently transfected with wild-type or mutated EGFR. We found that the mRNA level of *CLEC11A* expression was higher in H1299 cells with mutated EGFRs (H1299-L858R and Del) in comparison to those in the control cells (H1299-Vec) or cells with wild-type EGFR (H1299-EGFR). The differential expression of *CLEC11A* among H1299 derivatives was verified using both conventional and quantitative RT-PCR methods (Figure 1A). We also detected higher levels of CLEC11A proteins in lung cancer cell lines harboring an endogenous *EGFR* kinase domain mutation (Figure 1B). The expression of CLEC11A was only mildly suppressed by the addition of gefitinib, which evidently blocked EGFR phosphorylation, suggesting that *CLEC11A* expression might not be an immediate downstream effect of EGFR hyper-activation (Figure 1C).

**Figure 2:**
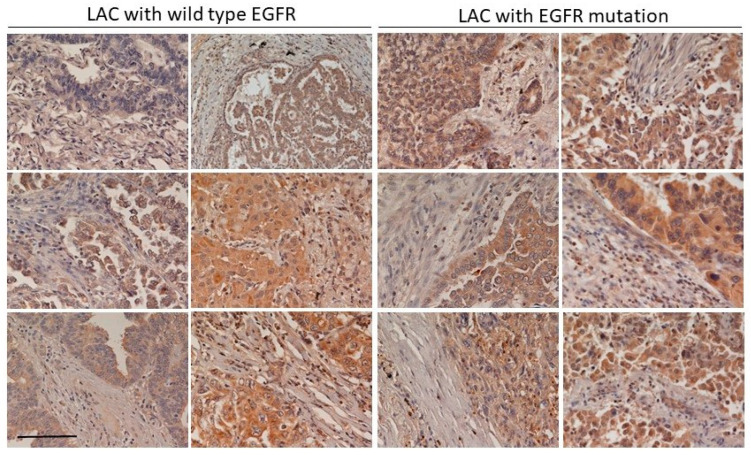
CLEC11A expression is frequently detected in LAC tissues with an *EGFR* mutation. Tissue sections from LAC with or without *EGFR* kinase domain mutations were examined by IHC staining using an anti-CLEC11A monoclonal antibody. The light staining patterns of the connective tissue within the tumor sections served as internal, negative controls. Scale bar: 0.2 mm.

We also examined CLEC11A expression in LAC tissues with or without *EGFR* mutation using immunohistochemical (IHC) staining. We found that although presented in both cases, the expression of CLEC11A occurred more frequently in the LAC tissues with an *EGFR* mutation (Figure 2). The analyses of the data deposited in the TCGA database also showed that LAC tissues had higher average *CLEC11A* expression levels in comparison with those in normal lung tissues, although without a statistical significance (Supplementary Figure S1A). Breaking down the LAC data in the TCGA based on the *EGFR* and *K-Ras* mutation status showed that *CLEC11A* expression levels are higher in tissues with an *EGFR* mutation than those with a *K-Ras* mutation or those without both (Supplementary Figure S1B). Collectively, our data indicated that CLEC11A expression was increased in LAC with an *EGFR* mutation in the kinase domain.

Additionally, we analyzed the correlation of CLEC11A levels and survival of LAC patients through the Kaplan–Meier plotter. The result showed that higher expression of *CLEC11A* was associated with a worse survival rate in Stage 1 LAC patients (Supplementary Figure S1C). In addition to LAC, CLEC11A expression levels were also higher in breast cancer (BRCA) and head and neck squamous carcinoma (HNSC) tissues based on the clinical data in the TCGA (Supplementary Figure S1D). Taken together, these results indicate that *CLEC11A* expression might be involved in the progression of various cancers.

### 3.2. CLEC11A Expression Promotes Tumor Formation

**Figure 3:**
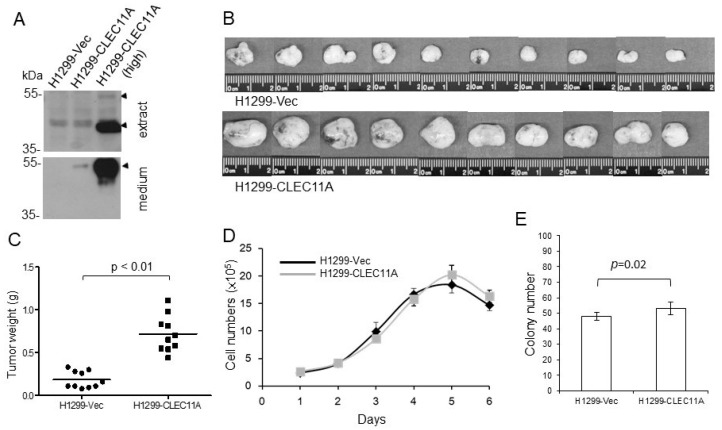
H1299 cells expressing CLEC11A have higher tumorigenic ability in vivo. (**A**) H1299-CLEC11A and CLEC11A (high) cells were established from permanently transfected cells. (**B**) H1299-Vec and H1299-CLEC11A cells were injected subcutaneously into nude mice. Tumors were harvested 5 weeks post-transplantation, weighed, and photographed. (**C**) Tumors formed by H1299-CLEC11A cells were significantly larger (Student *t*-test) than those formed by control cells. (**D**) H1299-Vec and H1299-CLEC11A cells were plated at 1 × 10^5^ cells/well and the cell numbers were measured at indicated time points. (**E**) H1299-Vec and H1299-CLEC11A cells were plated at 100 cells/6 cm plate and cultured for 2 weeks and were fixed and stained with crystal violet. The numbers of colonies were determined and the statistical analyses were performed by the Student *t*-test.

We established CLEC11A-overexpressing cells from the H1299 cell line, which expressed wild-type EGFR and a low level of CLEC11A (Figure 1B). Two populations of H1299 derivatives, H1299-CLEC11A and H1299-CLEC11A (high), were established with low and high levels of CLEC11A, respectively (Figure 3A). First, we tested the tumorigenic ability of H1299-CLEC11A in nude mice and found that CLEC11A expression significantly promoted the tumor formation by H1299 cells (Figure 3B,C). Interestingly, H1299-CLEC11A did not proliferate significantly faster than H1299-Vec control cells in regular cultures in vitro (Figure 3D). When seeding at low density, H1299-CLEC11A cells formed more colonies, statistically, than did the control cells in two weeks; however, the difference was minor (Figure 3E).

**Figure 4:**
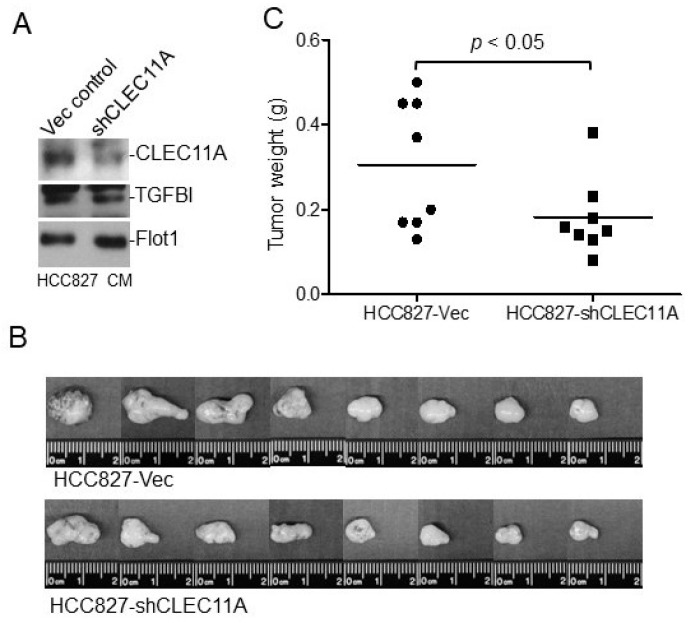
Suppression of CLEC11A expression in HCC827 cells decreases tumorigenic ability. (**A**) HCC827-Vec and HCC827-shCLEC11A cells were established by permanent transfection of pSuper-puromycin and pSuper-shCLEC11A vectors, respectively. The expression levels of the indicated proteins in the conditioned medium (CM) were examined by immune-blotting. (**B**) HCC827-Vec and HCC827-shCLEC11A cells were injected subcutaneously into nude mice. Tumors were harvested 5 weeks post-transplantation, weighed, and photographed. (**C**) Tumors formed by HCC827-shCLEC11A cells were significantly larger (Student *t*-test) than those formed by control cells.

To examine the importance of CLEC11A in the tumorigenicity of LAC with endogenous *EGFR* mutation, we established HCC827 cells with a decreased CLEC11A production through expressing a small hairpin RNA (HCC827-shCLEC11A). As shown in Figure 4A, the conditioned medium collected from the HCC827-shCLEC11A cells contained a lower level of CLEC11A, but not of TGFBI or Flotillin 1 (Flot1), in comparison to the control conditioned medium. We then tested the tumorigenic ability of HCC827-Vec and HCC827-shCLEC11A cells in nude mice and found that the reduction of CLEC11A significantly hampered the tumor formation by HCC827 cells (Figure 4B,C).

### 3.3. CLEC11A Promotes Tumor Angiogenesis In Vivo

**Figure 5:**
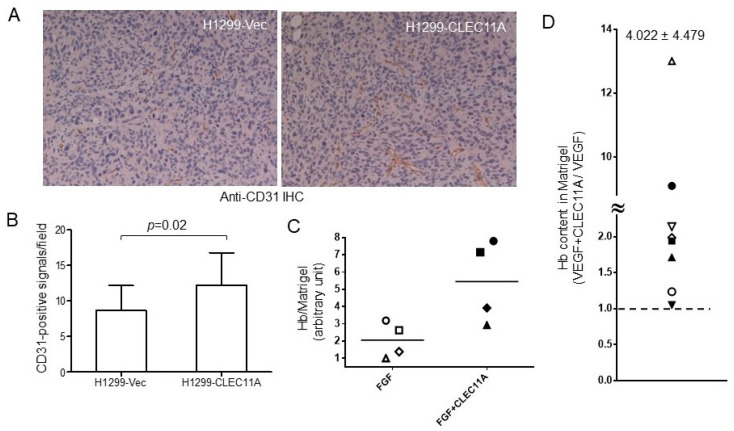
CLEC11A-expressing tumors contain more CD31-positive cells of endothelial lineage. (**A**) Tissue sections prepared from tumors formed by H1299-Vec and H1299-CLEC11A cells were subjected to IHC staining using an anti-CD31 antibody. (**B**) Images from the experiments of panel A were quantitated and the data were analyzed by Student *t*-test. (**C**) In vivo angiogenesis assays were performed as described in Materials and Methods. The levels of blood vessel content in Matrigel with FGF1 or FGF1 plus CLEC11A were quantitated by measuring the hemoglobin (Hb) levels. The symbols (open and closed) with the same shape represented the data collected from Matrigel blobs of the same mouse. (**D**) This experiment was performed as described in panel C, except using VEGF-A instead of FGF1. The data were presented as the ratios of Hb content in the Matrigel blob containing VEGF plus CLEC11A over that containing VEGF alone from the same mouse.

The apparent discrepancy between the significant tumor-promoting and minor cell growth-enhancing abilities of CLEC11A prompted us to look for the mechanism(s) that CLEC11A might have employed to enhance tumor formation in vivo. Since vascular formation is an important step for tumor progression, we examined the abundance of endothelial cells, one indicator of angiogenesis, in the xeno-transplanted tumors obtained above. Tumors with similar sizes were selected from both groups, and the presence of endothelial cells was examined by IHC staining using an antibody against CD31, an endothelial marker. We found that the tumors derived from H1299-CLEC11A cells contained significantly more CD31-positive cells than those formed by H1299-Vec cells, suggesting that the H1299-CLEC11A tumors had higher angiogenic activity (Figure 5A,B).

To examine whether the presence of exogenous CLEC11A is sufficient in promoting tumor growth and angiogenesis, we transplanted Lewis lung cancer cells, LL/2, into syngeneic C57BL/6 mice in the presence or absence of CLEC11A addition to the Matrigel matrix. We found that the addition of exogenous CLEC11A in the LL/2 transplantation was sufficient to promote tumor growth and to increase the number of endothelial cells in tumors (Supplementary Figure S2). Using an in vivo angiogenesis assay measuring blood vessel formation into Matrigel plugs, we found that CLEC11A alone did not induce significant angiogenesis (data not shown). However, the combination of CLEC11A with an angiogenic factor, either basic FGF or VEGF-A, induced better vessel formation than the angiogenic factor alone (Figure 5C,D). Collectively, these data suggested that CLEC11A might promote tumor growth through enhancing angiogenesis.

### 3.4. CLEC11A Has Direct Effects on Mouse Endothelial Cells

CLEC11A has been shown to promote endothelial cell differentiation from their precursor cells [8]. The increase of endothelial lineage cells in CLEC11A-expressing tumors could be the result of this function. Another possibility was that the presence of CLEC11A induced the migration of endothelial cells toward tumor tissues. We tested the chemo-attractive ability of CLEC11A against the mouse endothelial cell line SVEC4-10 and found that conditioned medium collected from H1299-CLEC11A cells did promote migration of SVEC4-10 cells better than that of H1299 control cells (Supplementary Figure S3A). The chemo-attractant ability in the condition medium was suppressed by an anti-CLEC11A antibody (Supplementary Figure S3B).

**Figure 6:**
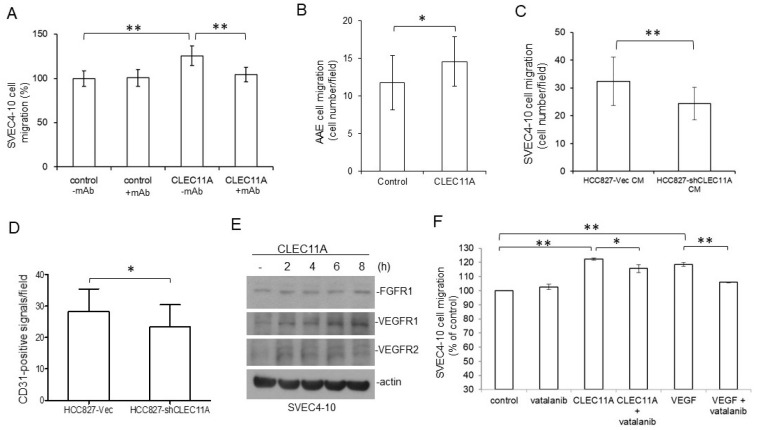
CLEC11A is a factor with angiogenic potential. (**A**) The chemo-attractant ability of purified CLEC11A (30 ng/mL) was tested against SVEC4-10 cells in the presence or absence of the anti-CLEC11A monoclonal antibody (1 μg/mL) in the Transwell chambers. (**B**) The chemo-attractant ability of purified CLEC11A (30 ng/mL) was tested against primary AAE cells in the Transwell chambers. (**C**) Conditioned media of HCC827-Vec and HCC827-shCLEC11A cells were tested for their chemo-attractant ability against SVEC4-10 cells. (**D**) Tissue sections prepared from tumors formed by HCC827-Vec and HCC827-shCLEC11A cells were subjected to IHC staining using an anti-CD31 antibody. Staining images were quantitated and the data were analyzed by the Student *t*-test. (**E**) Overnight 1% serum-starved SVEC4-10 cells were treated with 30 ng/mL of CLEC11A for the indicated times and the levels proteins of interest were examined by immune-blotting. (**F**) The chemo-attractant activities of CLEC11A (30 ng/mL) or VEGF-A (20 ng/mL) were tested against SVEC4-10 cells in the presence or absence of vatalanib (5 mM) in the Transwell chambers. Statistical significances between treatments are presented as single asterisk (*p* < 0.05) or double asterisks (*p* < 0.01).

To prove that CLEC11A per se had the chemo-attracting ability on endothelial cells, CLEC11A affinity purified from H1299-CLEC11A (high) conditioned medium was tested against SVEC4-10 cells. We found that purified CLEC11A attracted SVEC4-10 cell migration, and that the monoclonal antibody against CLEC11A blocked the cell migration induced by CLEC11A, while the monoclonal antibody itself did not affect the migration of the SVEV4-10 cells (Figure 6A). Similarly, primary aortic artery endothelial cells (AAE cells) also displayed an increase of migration ability in the presence of purified CLEC11A (Figure 6B).

Consistent with the data in Figure 5A,B, the conditioned medium collected from HCC827-shCLEC11A cells showed less ability in attracting the migration of SVEC4-10 cells (Figure 6C). When examined by immunohistochemical (IHC) staining, we found that tumors derived from HCC827-shCLEC11A cells contained less CD31-positive cells than those formed by HCC827-Vec cells (Figure 6D). These data indicated that the suppression of CLEC11A production in HCC827 cells decreased the tumorgenicity of this cell line.

Interestingly, we found that the treatment of SVEC4-10 cells with CLEC11A increased the expression of FGFR1 as well as VEGFR1 and 2 in the SVEC4-10 cells (Figure 6E). This observation was in agreement with the combinatory effects of CLEC11A and VEGF-A (or basic FGF) in vivo (Figure 5C,D), suggesting that CLEC11A might have acted on the endothelial cells through promoting FGF and/or VEGF signaling. However, we found that vatalanib, a VEGFR-selective inhibitor, only partially inhibited CLEC11A-induced cell migration, while the same dose of vatalanib almost completely abolished VEGF-A-induced SVEC4-10 cell migration (Figure 6F). This result indicated that the chemo-attractant effect of CLEC11A on endothelial cells might be not thoroughly mediated through the expression of receptors for endothelial tropic growth factors. Our data indicated that CLEC11A had direct biochemical and biological effects on endothelial cells.

### 3.5. CLEC11A Acts through Integrins but Does Not Induce Conventional Integrin Signaling

**Figure 7:**
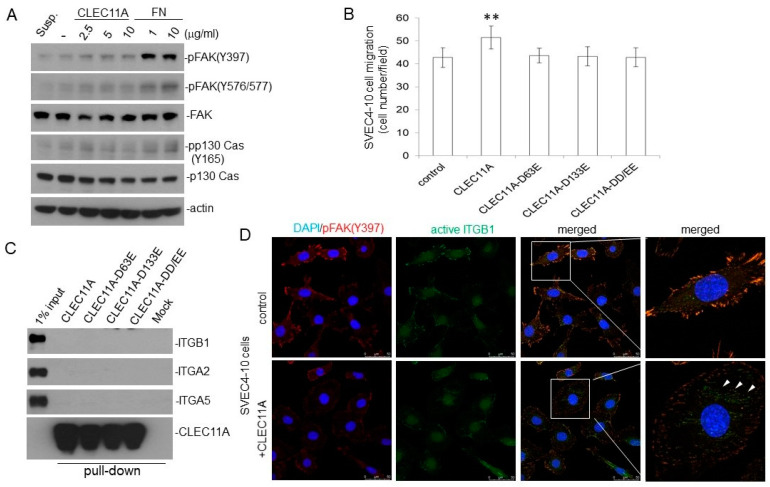
Integrin receptors are involved in CLEC11A action on endothelial cells. (**A**) SVEC4-10 cells were plated on CLEC11A (2.5–10 μg/mL) or fibronectin (FN; 1 or 10 μg/mL)-coated plates for 20 min. The levels of indicated proteins were determined by immune-blotting. (**B**) Wild-type and different mutant CLEC11A proteins (30 ng/mL) were tested for their chemo-attractant ability against SVEC4-10 cells. ** *p* < 0.01. (**C**) His-tagged wild-type and different mutant CLEC11A proteins were immobilized on Co^2+^-conjugated agarose beads and were incubated with SVEC4-10 lysates for testing of their integrin-binding ability. The levels of indicated proteins in the complexes were determined by immune-blotting. (**D**) Overnight 1% serum-starved SVEC4-10 cells were treated with or without CLEC11A (30 ng/mL) for 20 min. The cells were then fixed and subjected to immuno-fluorescence staining using antibodies against pFAK (Y397) and active integrin β1 (ITGB1). DAPI was used to stain cell nuclei. Arrow heads indicate the signals of active ITGB1.

CLEC11A has been shown to promote osteogenesis through binding to integrin α11β1 on osteoblasts [10]. Therefore, we examined whether CLEC11A also utilized the integrin signaling pathway in endothelial cells. We found that, different from fibronectin, CLEC11A failed to induced significant Tyr phosphorylation of focal adhesion kinase (FAK) and p130 Cas in SVEC4-10 cells (Figure 7A). However, mutations of potential integrin-binding motifs (RGD and/or LDT) on CLEC11A did abolish the chemo-attractant ability of this factor against SVEC4-10 cells (Figure 7B), suggesting a biological function(s) of these motifs. We did not observe an interaction between His-tagged CLEC11A with integrin components (ITGs β1, α2, and α5) expressed in SVEC4-10 cells using the in vitro pull-down assays (Figure 7C). However, we observed an increase of the active integrin β1 signal, detected by a conformation-specific antibody, upon CLEC11A treatment in SVEC4-10 cells. Nevertheless, the newly appeared foci of active integrin β1 were not associated with active FAK (pFAK) in CLEC11A-treated cells (Figure 7D). Taken together, these results suggested that CLEC11A acts on endothelial cells through integrins, but does not induce conventional integrin signaling significantly.

## 4. Discussion

The formation of tumor vasculature for blood supply is achieved through multiple mechanisms, including (but not limited to) (1) the development of blood vessels from endothelial cells (ECs) in pre-existing vessels (angiogenesis), (2) the formation of vessels by EC precursors (vasculogenesis), (3) tumor cells growing along the existing vasculature (vessel co-option), and (4) the formation of vessel-like structures by tumor cells (vasculogenic mimicry) [1,2,19]. Angiogenic factors may participate in most of these processes [1,19]. Anti-angiogenic therapeutics, either monoclonal antibody- or chemical-based, against endothelial trophic factors/receptors have been developed for years. However, anti-angiogenic therapy alone is usually not sufficient to achieve a satisfactory effect, and combination with conventional chemotherapy is required for most of the cancer-treating regiments [1]. The complex mechanisms that solid tumors utilize to evade blockades and achieve blood supply may provide explanations for the current problems in anti-angiogenic therapy. Another possible explanation is that multiple factors (and receptors) are involved in the process of tumor angiogenesis. Although the concept of stopping blood flow to tumors is straightforward, the blocking of a single pathway with a specific antibody or inhibitor proved insufficient in achieving the desired effect. Therefore, a better understanding of the angiogenic mechanisms involved in tumor progression is very important.

Our findings suggested that CLEC11A produced from LAC may promote tumor vascularization and cancer progression. CLEC11A per se did not show angiogenic activity, since the factor alone could not support significant blood vessel formation in the in vivo angiogenesis assay (data not shown). However, the combination of CLEC11A and VEGF-A (or FGF1) achieved better angiogenic effects than did VEGF-A (or basic FGF) alone (Figure 5D,E). The mechanism by which CLEC11A promoted angiogenesis was intriguing. CLEC11A promotes endothelial cell differentiation, in the presence of VEGF-A, from AC133-positive progenitor cells [8]. It is possible that CLEC11A secreted from LAC cells facilitated the differentiation of circulating progenitor cells into endothelial cells and the formation of new vessels through vasculogenesis, in the presence of tumor-secreted VEGFs. We found that CLEC11A increased the expression levels of FGFR1 and VEGFRs in SVEC4-10 endothelial cells (Figure 6E). It was also possible that the presence of CLEC11A triggered the better responses of endothelial cells to angiogenic growth factors. Additionally, our data indicated that CLEC11A is a chemo-attractant for mouse endothelial cells (Figure 6). This chemo-attractant ability of CLEC11A was not dependent on the VEGF/VEGFR signaling, since the VEGFR inhibitor vatalanib did not abolish this effect (Figure 6F). It was likely that cancer cells secreted CLEC11A and attracted endothelial cells sprouting from the nearby pre-existing blood vessels and, subsequently, forming new blood vessels in the growing tumor mass. Our data indicated that understanding the biological effect of CLEC11A could be very important in achieving a better understanding of tumor angiogenesis.

Despite the fact that CLEC11A has been identified for more than three decades, we do not understand much about the biological functions of CLEC11A and how those functions link to its structural configuration. A major difference between human and mouse CLEC11A is that the mouse protein does not contain an RGD motif [6,10]. This motif, along with the LDT motif, is proposed as the binding motifs on CLEC11A to integrin receptors [10]. We found that CLEC11A mutated at the RGD and/or LDT motifs lost the chemo-attractant ability toward endothelial cells, suggesting that these motifs, directly or indirectly, were involved in this function (Figure 7B). Contrary to the previous report, we did not observe the physical interaction between CLEC11A and integrin receptors (Figure 7C). We also did not detect significant activation of FAK in the endothelial cells by CLEC11A stimulation, unlike fibronectin (Figure 7A). Nevertheless, we detected the activation of integrin β1 by CLEC11A using a conformation-specific antibody (Figure 7D). Our collective data suggested that the integrin(s) might participate in the biological effects of CLEC11A in endothelial cells, but conventional integrin signaling may not be important. It should be noted that the promoting effects of CLEC11A on osteoblast differentiation were observed in assays using human, not mouse, CLEC11A [9,10]. It would be interesting to know whether the RGD and LDT motifs are essential for all of the biological effects of CLEC11A.

We found that the increase of CLEC11A in LAC was more common in LAC with *EGFR* mutation (Figure 1 and Figure 2). Mutations of EGFR at its kinase domain, which frequently occur in LAC patients in east Asian populations, are associated with the clinical responsiveness to EGFR tyrosine kinase inhibitors (TKIs) [20,21]. Despite the initial effectiveness of TKIs in treating LAC with *EGFR* mutation, drug resistance frequently develops within one year [22]. New therapeutic approaches remain desirable for the better treatment of LAC patients [23]. Interestingly, gefitinib failed to effectively abolish CLEC11A expression in LAC cells with *EGFR* mutation, suggesting that CLEC11A was not induced by signaling immediately downstream of the mutated EGFR (Figure 1C). The EGFR protein is capable of achieving some biological functions independent of its kinase activity [24]. EGFR itself can also localize to nuclei and serve as a transcriptional factor [25]. It will be interesting to know whether wild-type and mutated EGFR proteins are different in these aspects. Would it be possible for CLEC11A to be targeted for cancer therapeutic purposes? The initial studies on CLEC11A (previously named SCGF) implicated its role in the survival of leukemia cells [5]. The neutralization antibody against CLEC11A induces cell death in multiple leukemia cell lines [26]. However, later studies did not support the biological function of CLEC11A in regulating hematopoiesis [9]. Several studies have reported the increase of CLEC11A in various malignant diseases, but the pathological effect of this increase remains unclear [27,28]. From the clinical information in the TCGA database, we found that a significant increase of CLEC11A also occurred in other types of carcinomas, such as head and neck cancer and breast cancer (Supplementary Figure S1). It is possible that the increase of CLEC11A is a common feature in the progression of various cancers. Since CLEC11A-knockout mice did not show significant defects in the hematopoietic system, and the most significant phenotype observed in CLEC11A-null mice is the defect in bone formation in vertebrates and limb bones, the mice could survive without CLEC11A [9]. It is likely that the loss of CLEC11A is bearable for a living organism, and that targeting the CLEC11A over-produced by cancers is a potential treatment strategy. The evidence provided in our study warrants further studies of the role of CLEC11A in tumor angiogenesis and the potential of targeting CLEC11A for cancer therapy.

## 5. Conclusions

In summary, our study showed that CLEC11A expression is increased in lung cancer cell lines and lung cancer tissues harboring mutated EGFR. CLEC11A promotes higher tumorigenic ability. In contrast, the suppression of CLEC11A expression decreases the tumorigenic ability. CLEC11A has angiogenic potential, because CLEC11A promotes the angiogenesis induced by VEGF and FGF. Integrin receptors may be involved in CLEC11A’s action on endothelial cells.

## Data Availability

The data presented in this study are available in this article and in supplemental material.

## Supplementary Materials

The following are available online at https://www.mdpi.com/article/10.3390/cancers14051356/s1: Supplementary Figure Legend, Figure S1: CLEC11A expression is higher in LAC tissues with *EGFR* mutation; Figure S2: Addition of CLEC11A promotes tumor formation and recruitment of endothelial cells by LL/2 cells; Figure S3: The conditioned medium produced by H1299-CLEC11A cells have chemo-attractant ability against SEVC4-10 endothelial cells.

## References

[1] Bergers G., Benjamin L.E. (2003). Tumorigenesis and the angiogenic switch. Nat. Rev. Cancer.

[2] Aguilar-Cazares D., Chavez-Dominguez R., Carlos-Reyes A., Lopez-Camarillo C., de la Cruz O.N.H., Lopez-Gonzalez J.S. (2019). Contribution of angiogenesis to inflammation and cancer. Front. Oncol..

[3] Harris A.L. (2001). Hypoxia—A key regulatory factor in tumour growth. Nat. Rev. Cancer.

[4] Rivas-Fuentes S., Salgado-Aguayo A., Pertuz Belloso S., Gorocica Rosete P., Alvarado-Vásquez N., Aquino-Jarquin G. (2015). Role of chemokines in non-small cell lung cancer: Angiogenesis and inflammation. J. Cancer.

[5] Hiraoka A., Ohkubo T., Fukuda M. (1987). Production of human hematopoietic survival and growth factor by a myeloid leukemia cell line (KPB-M15) and placental as detected by a monoclonal antibody. Cancer Res..

[6] Mio H., Kagami N., Yokokawa S., Kawai H., Nakagawa S., Takeuchi K., Sekine S., Hiraoka A. (1998). Isolation and characterization of a cDNA for human, mouse, and rat full-length stem cell growth factor, a new member of C-type lectin superfamily. Biochem. Biophys. Res. Comm..

[7] Bannwarth S., Giordanengo V., Lesimple J., Lefebvre J.-C. (1998). Molecular cloning of a new secreted sulfated mucin-like protein with a C-type lectin domain that Is expressed in lymphoblastic cells. J. Biol. Chem..

[8] Gehling U.M., Ergün S., Schumacher U., Wagener C., Pantel K., Otte M., Schuch G., Schafhausen P., Mende T., Kilic N. (2000). In vitro differentiation of endothelial cells from AC133-positive progenitor cells. Blood.

[9] Yue R., Shen B., Morrison S.J. (2016). Clec11a/osteolectin is an osteogenic growth factor that promotes the maintenance of the adult skeleton. eLife.

[10] Shen B., Vardy K., Hughes P., Tasdogan A., Zhao Z., Yue R., Crane G.M., Morrison S.J. (2019). Integrin alpha11 is an Osteolectin receptor and is required for the maintenance of adult skeletal bone mass. eLife.

[11] Schiller J.H., Bittner G. (1995). Loss of the tumorigenic phenotype with in vitro, but not in vivo, passaging of a novel series of human bronchial epithelial cell lines: Possible role of an alpha 5/beta 1-integrin-fibronectin interaction. Cancer Res..

[12] Walter-Yohrling J., Morgenbesser S., Rouleau C., Bagley R., Callahan M., Weber W., Teicher B.A. (2004). Murine endothelial cell lines as models of tumor endothelial cells. Clin. Cancer Res..

[13] Shiao Y.-M., Chang Y.-H., Liu Y.-M., Li J.-C., Su J.-S., Liu K.-J., Liu Y.-F., Lin M.-W., Tsai S.-F. (2008). Dysregulation of GIMAP genes in non-small cell lung cancer. Lung Cancer.

[14] Yang C.-H., Chou H.-C., Fu Y.-N., Yeh C.-L., Cheng H.-W., Chang I.-C., Liu K.-J., Chang G.-C., Tsai T.-F., Tsai S.-F. (2015). EGFR over-expression in non-small cell lung cancers harboring EGFR mutations is associated with marked down-regulation of CD82. BBA Mol. Basis Dis..

[15] Chen Y.-R., Fu Y.-N., Lin C.-H., Yang S.-T., Hu S.-F., Chen Y.-T., Tsai S.-F., Huang S.-F. (2006). Distinctive activation patterns in constitutively active and gefitinib-sensitive EGFR mutants. Oncogene.

[16] Kobayashi M., Inoue K., Warabi E., Minami T., Kodama T. (2005). A simple method of isolating mouse aortic endothelial cells. J. Atheroscler. Thromb..

[17] Chen Y.-R., Chou H.-C., Yang C.-H., Chen H.-Y., Liu Y.-W., Lin T.-Y., Yeh C.-L., Chao W.-T., Tsou H.-H., Chuang H.-C. (2017). Deficiency in VHR/DUSP3, a suppressor of focal adhesion kinase, reveals its role in regulating cell adhesion and migration. Oncogene.

[18] Huang S.-F., Liu H.-P., Li L.-H., Ku Y.-C., Fu Y.-N., Tsai H.-Y., Chen Y.-T., Lin Y.-F., Chang W.-C., Kuo H.-P. (2004). High frequency of epidermal growth factor receptor mutations with complex patterns in non-small cell lung cancer related to gefitinib responsiveness in Taiwan. Clin. Cancer Res..

[19] De la Cruz O.N.H., López-González J.S., García-Vázquez R., Salinas-Vera Y.M., Muñiz-Lino M.A., Aguilar-Cazares D., López-Camarillo C., Carlos-Reyes Á. (2020). Regulation networks driving vasculogenic mimicry in solid tumors. Front. Oncol..

[20] Paez J.G., Jänne P.A., Lee J.C., Tracy S., Greulich H., Gabriel S., Herman P., Kaye F.J., Lindeman N., Boggon T.J. (2004). EGFR mutations in lung cancer: Correlation with clinical response to gefitinib therapy. Science.

[21] Lynch T.J., Bell D.W., Sordella R., Gurubhagavatula S., Okimoto R.A., Brannigan B.W., Harris P.L., Haserlat S.M., Supko J.G., Haluska F.G. (2004). Activating mutations in the epidermal growth factor receptor underlying responsiveness of non-small cell lung cancer to gefitinib. N. Engl. J. Med..

[22] Kobayashi S., Boggon T.J., Dayaram T., Janne P.A., Kocher O., Meyerson M., Johnson B.E., Eck M.J., Tenen D.G., Halmos B. (2005). EGFR mutations and resistance of non-small-cell lung cancer to gefitinib. N. Engl. J. Med..

[23] Riely G.J. (2008). Second-generation epidermal growth factor receptor tyrosine kinase inhibitors in non-small cell lung cancer. J. Thorac. Oncol..

[24] Weihua Z., Tsan R., Huang W.-C., Wu Q., Chiu C.-H., Fidler I.J., Hung M.-C. (2008). Survival of cancer cells is maintained by EGFR independent of its kinase activity. Cancer Cell.

[25] Lin S.-Y., Makino K., Xia W., Matin A., Wen Y., Kwong K.Y., Bourguignon L., Hung M.-C. (2002). Nuclear localization of EGF receptor and its potential new role as a transcription factor. Nat. Cell Biol..

[26] Hiraoka A. (2008). Leukemia cell lines require self-secreted stem cell growth factor (SCGF) for their proliferation. Leuk. Res..

[27] Comara M., Zanottaa N., Zanconatic F., Cortaled M., Bonottie A., Cristaudoe A., Bovenzi M. (2016). Chemokines involved in the early inflammatory response and inpro-tumoral activity in asbestos-exposed workers from an Italiancoastal area with territorial clusters of pleural malignant mesothelioma. Lung Cancer.

[28] Riva L.D., Bozzi F., Mondellini P., Miccichè F., Fumagalli E., Vaghi E., Huber V., Gronchi A., Tamborini E., Pierotti M.A. (2011). Proteomic detection of a large amount of SCGFa in the stroma of GISTs after imatinib therapy. J. Transl. Med..

