# Supplementary material for: EGFR Mutation-Harboring Lung Cancer Cells Produce CLEC11A with Endothelial Trophic and Tumor-Promoting Activities
Source: Cancers (Basel). 2022 Mar 7;14(5):1356. doi: 10.3390/cancers14051356 (PMC8909374; doi:10.3390/cancers14051356)
Supplement: Supplementary file 1 [file cancers-14-01356-s001.zip › cancers-1621656 supplementary materials.pdf]

Supplementary Materials: *EGFR* Mutation-Harboring Lung Cancer Cells Produce CLEC11A with Endothelial Trophic and Tumor-Promoting Activities

Tzu-Yin Lin, Chi-Hwa Yang, Hsiao-Chin Chou, Chun-Mei Cheng, Ya-Wen Liu [1], Jiz-Yuh Wang, Li-Rung Huang, Shih-Feng Tsai, Shiu-Feng Huang and Yi-Rong Chen

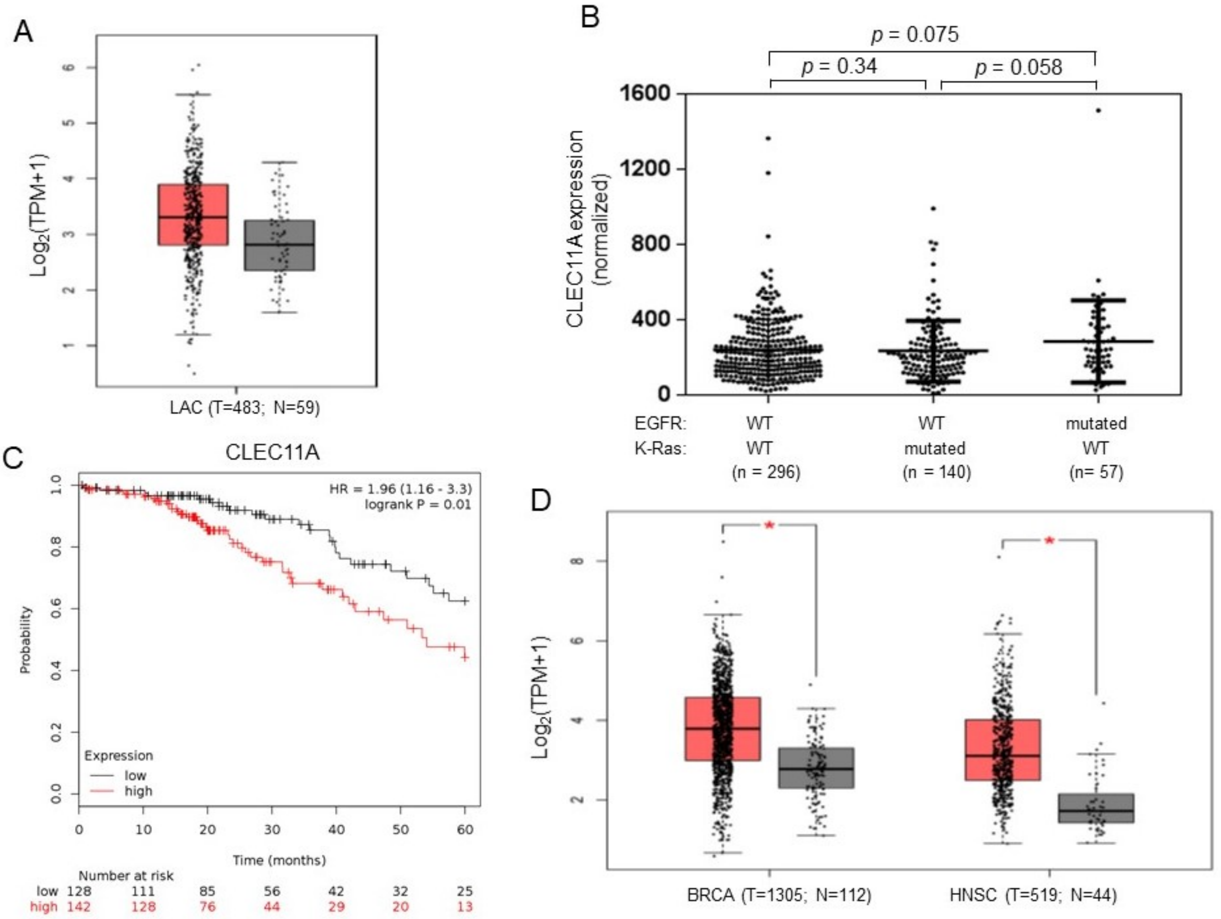

Figure S1. CLEC11A expression is higher in LAC tissues with *EGFR* mutation. (**A**) Data of LAC and normal lung in TCGA database (Red: tumor/T; Grey: normal/N) were analyzed for *CLEC11A* mRNA levels (TPM, transcript per million) through the Boxplot grogram in Gene Expression Profiling Interactive Analysis (GEPIA). (**B**) CLEC11A expression levels of LAC patients were retrieved through Oncolnc and were sub-grouped according to the presence or absence of *EGFR* and *K-Ras* mutation in the Genomic Data Commons (GDC) data portal, NCI. (**C**) The correlation between CLEC11A levels (RNAseq) and survival of LAC patients was examined through the Kaplan Meier plotter with auto selected best cutoff. (D) Data of breast cancer (BRCA) and head and neck cancer (HNSC) in TCGA database were analyzed for *CLEC11A* levels through the Boxplot grogram in GEPIA.

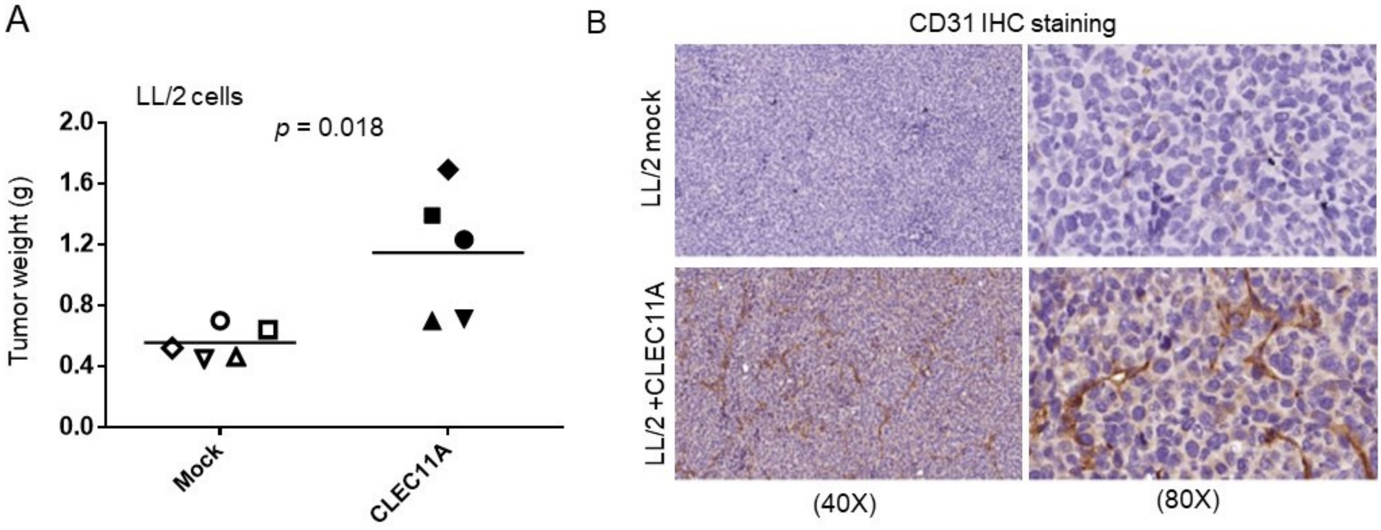

Figure S2. Addition of CLEC11A promotes tumor formation and recruitment of endothelial cells by LL/2 cells. (**A**) One million LL/2 lung cancer cells were mixed in Matrigel either with or without hCLEC11A (1 mg/mL) and were injected subcutaneously into both franks of individual nude mice. Tumors were harvested 2 wks post-transplantation and weighted. Symbols of the same shapes represented tumors derived from the same mice. (**B**) Tissue sections prepared from tumors formed by LL/2 in the presence or absence of CLEC11A were subjected to IHC staining using an anti-CD31 antibody.

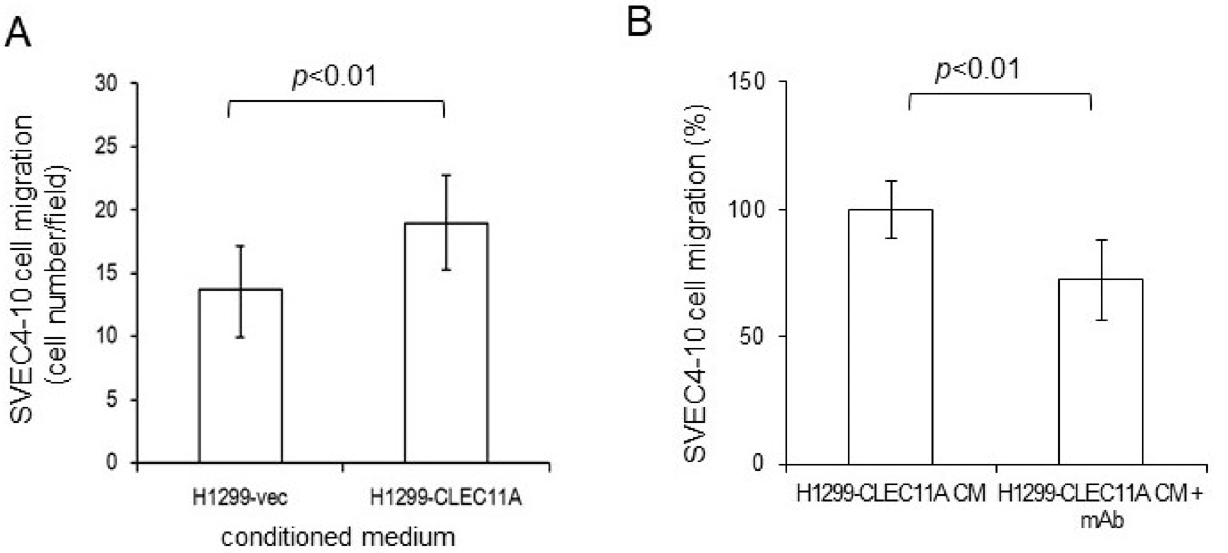

Figure S3. The conditioned medium produced by H1299-CLEC11A cells have chemo-attractant ability against SEVC4-10 endothelial cells. (**A**) Chemo-attractant activities of conditioned media collected from H1299-Vec or H1299-CLEC11A cells were tested against SVEC4-10 cells in the Transwell chambers as described in Materials and Methods. The data presented are the mean and standard deviations of a representative experiments. (**B**) Chemo-attractant activity of H1299-CLEC11A conditioned medium was tested in the presence or absence of an anti-CLEC11A monoclonal antibody (mAb; 1 µg/mL) against SVEC4-10 cells in the Transwell chambers. The data presented are the mean and standard deviations of a representative experiments.

Uncropped immunoblots

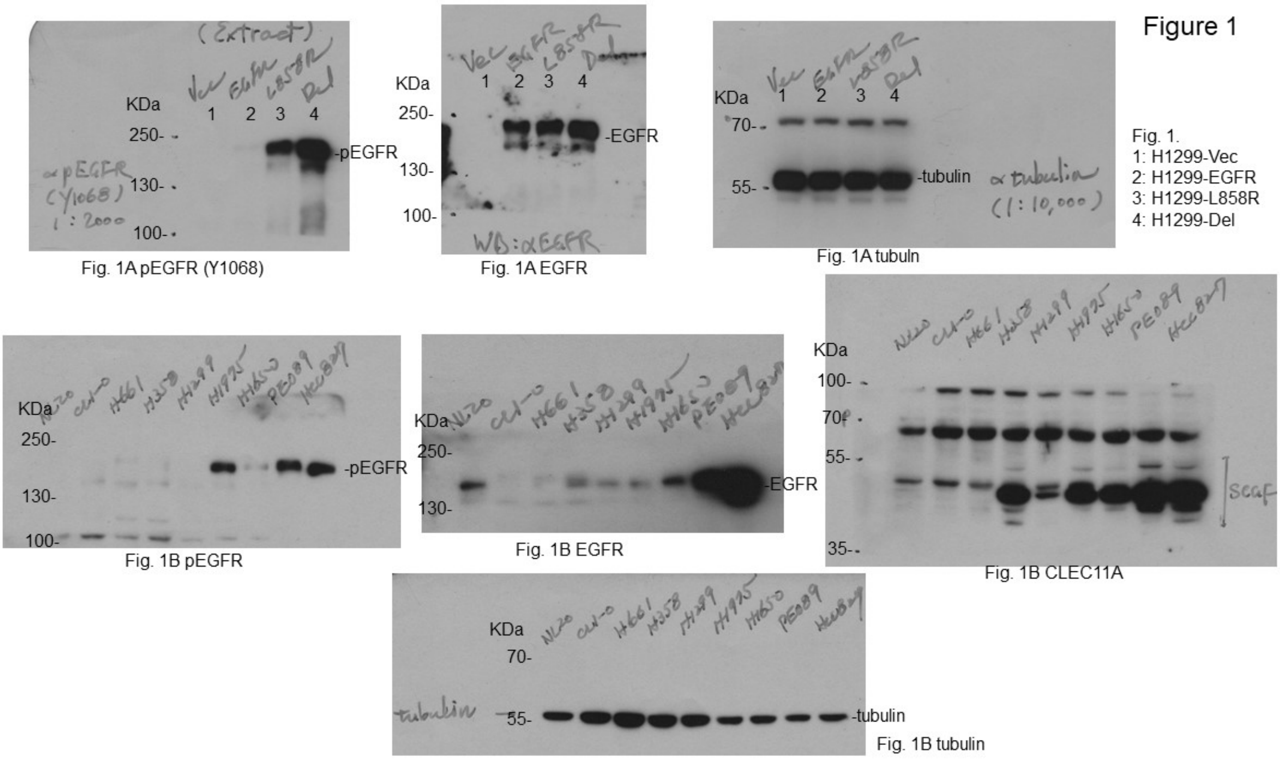

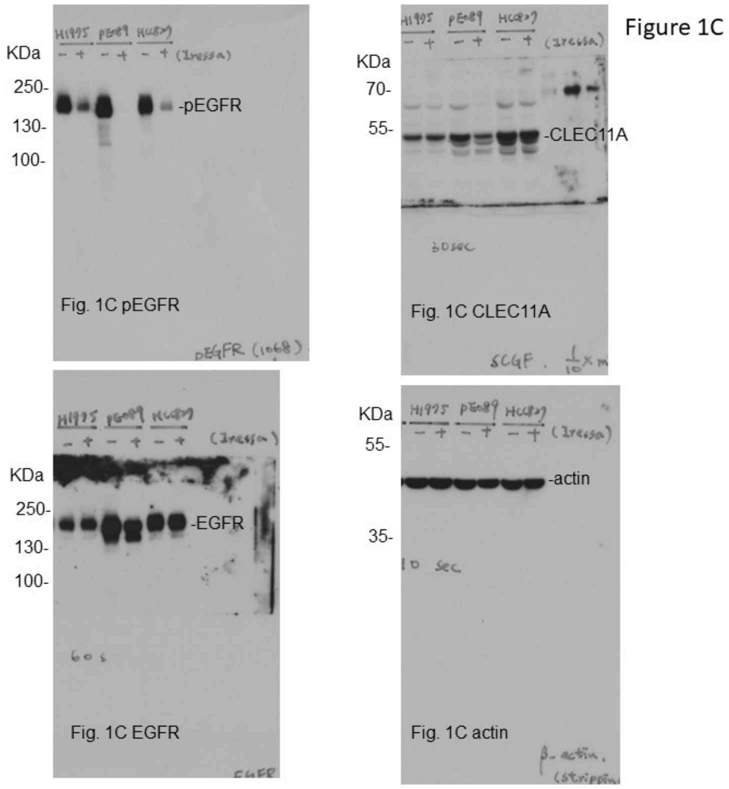

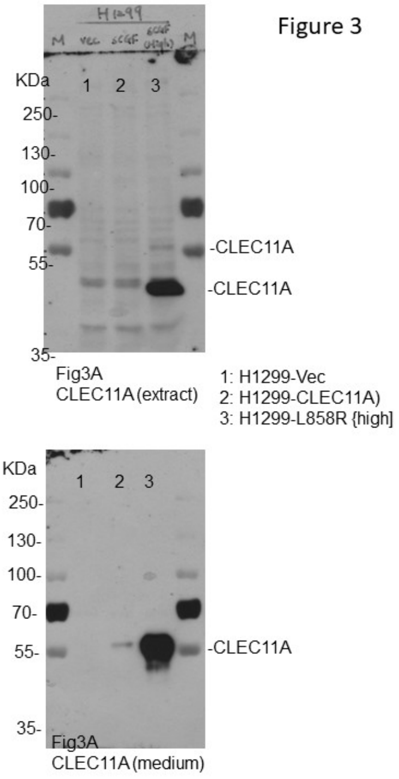

Uncropped immunoblots

Figure 4

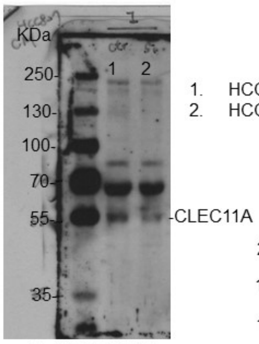

1. HCC827-Vec-CM
2. HCC827-shCLEC11A-CM

Fig4A CLEC11A

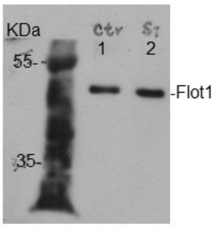

Fig4A Flot1

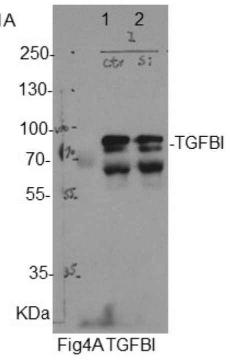

Fig4A TGFBI

Figure 6

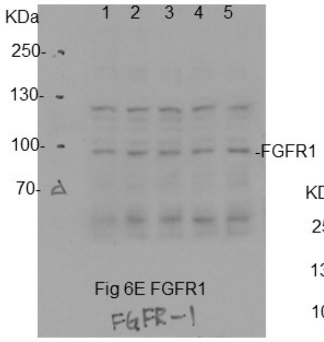

Fig 6E FGFR1

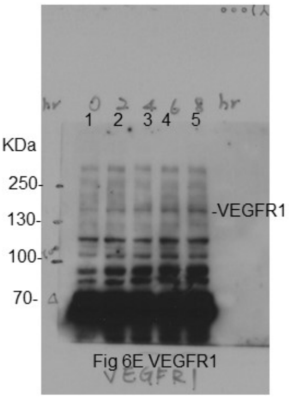

Fig 6E VEGFR1

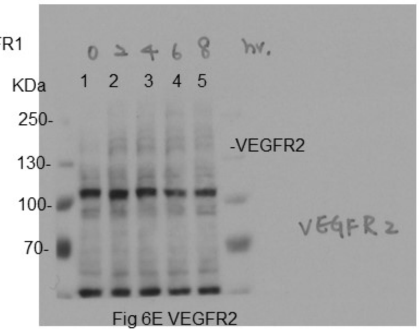

Fig 6E VEGFR2

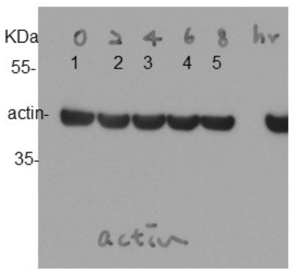

Fig 6E actin

SVEC4-10 treated with CLEC11A
1: 0 h
2: 2 h
3: 4 h
4: 6 h
5: 8 h

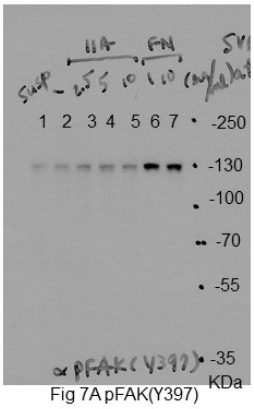

Fig 7A pFAK(Y397)

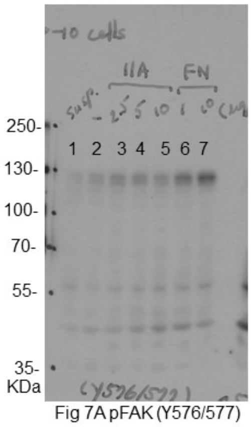

Fig 7A pFAK (Y576/577)

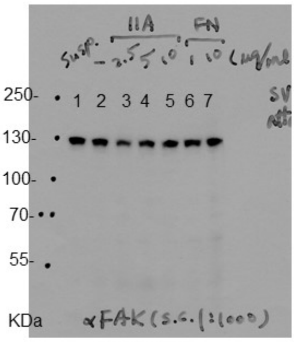

Fig 7A FAK

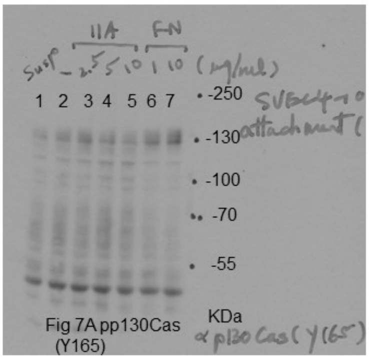

Fig 7A pp130Cas (Y165)

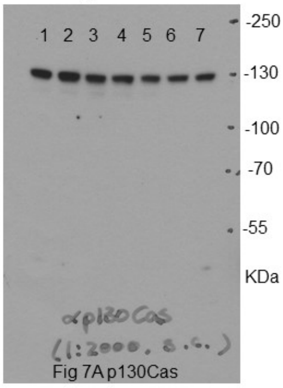

Fig 7A p130Cas

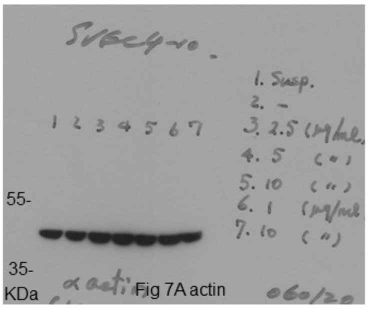

Fig 7A actin

Figure 7A

1. Suspended SVEC cells
2. Attached to non-coated
3. Attached to 2.5 μg/ml CLEC11A
4. Attached to 5 μg/ml CLEC11A
5. Attached to 10 μg/ml CLEC11A
6. Attached to 1 μg/ml fibronectin
7. Attached to μg/ml CLEC11A

Uncropped immunoblots

Figure 7C

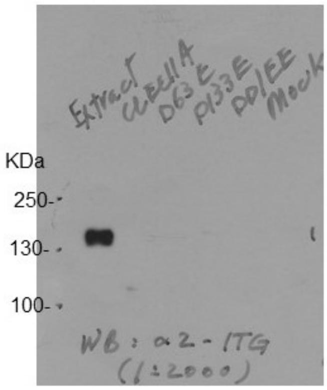

Fig 7C ITGA2

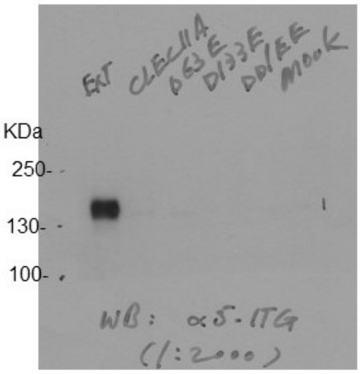

Fig 7C ITGA5

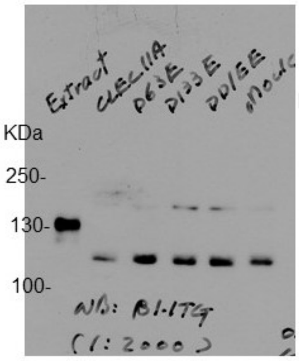

Fig 7C ITGB1

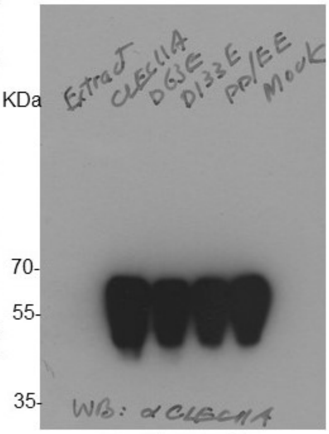

Fig 7C CLEC11A
